# The Effects of Age on Prostatic Responses to Oxytocin and the Effects of Antagonists

**DOI:** 10.3390/biomedicines11112956

**Published:** 2023-11-01

**Authors:** Masroor Badshah, Jibriil Ibrahim, Nguok Su, Penny Whiley, Michael Whittaker, Betty Exintaris

**Affiliations:** 1 Hudson Institute of Medical Research, Monash University, Clayton, VIC 3168, Australia; penny.whiley@hudson.org.au; 2 Drug Discovery Biology, Monash Institute of Pharmaceutical Sciences, Parkville, VIC 3052, Australiaeunice.su@monash.edu (N.S.); 3 Drug, Delivery, Disposition and Dynamics, Monash Institute of Pharmaceutical Sciences, Parkville, VIC 3052, Australia; michael.whittaker@monash.edu

**Keywords:** BPH, LUTS, oxytocin, oxytocin receptor antagonists

## Abstract

Benign prostatic hyperplasia (BPH) is an age-related enlargement of the prostate with urethral obstruction that predominantly affects the middle-aged and older male population, resulting in disruptive lower urinary tract symptoms (LUTS), thus creating a profound impact on an individual’s quality of life. The development of LUTS may be linked to overexpression of oxytocin receptors (OXTR), resulting in increased baseline myogenic tone within the prostate. Thus, it is hypothesised that targeting OXTR using oxytocin receptor antagonists (atosiban, cligosiban, and β-Mercapto-β,β-cyclopentamethylenepropionyl1, O-Me-Tyr2, Orn8]-Oxytocin (ßMßßC)), may attenuate myogenic tone within the prostate. Organ bath and immunohistochemistry techniques were conducted on prostate tissue from young and older rats. Our contractility studies demonstrated that atosiban significantly decreased the frequency of spontaneous contractions within the prostate of young rats (**** *p* < 0.0001), and cligosiban (* *p* < 0.05), and ßMßßC (**** *p* < 0.0001) in older rats. Additionally, immunohistochemistry findings revealed that nuclear-specific OXTR was predominantly expressed within the epithelium of the prostate of both young (*** *p* < 0.001) and older rats (**** *p* < 0.0001). In conclusion, our findings indicate that oxytocin is a key modulator of prostate contractility, and targeting OXTR is a promising avenue in the development of novel BPH drugs.

## 1. Introduction

Benign prostatic hyperplasia (BPH) is mainly a histological diagnosis characterised by the hyperplasia of stromal and glandular epithelial cells within the transition zone of the prostate [1]. Globally, the prevalence of BPH varies from 20–62% in *males* over the age of 50 years [2]. This condition begins as simple micronodular hyperplasia, which grows as a macroscopic nodular enlargement, eventually causing bladder outlet obstruction [3,4]. As a result of an enlarged prostate gland with an increase in smooth muscle tone, there is obstruction of the urethra that results in lower urinary tract symptoms (LUTS), such as urinary retention, detrusor instability, haematuria, bladder outlet obstruction, and renal insufficiency [5,6].

The exact cause of BPH is not known, but certain factors such as age and androgens, particularly dihydrotestosterone (DHT), participate in the development of BPH [7,8,9,10]. This DHT is formed from testosterone (androgen) in the presence of an enzyme known as 5-α reductase. Both testosterone and DHT bind to the nuclear-specific androgen receptor (AR), but DHT has 10 times higher affinity than testosterone [11]. The 5-α reductase inhibitors (finasteride and dutasteride) mainly inhibit the 5-α reductase enzyme, thus preventing the conversion of testosterone to DHT. This then results in reducing the size of the prostate, thus improving the urinary flow rate [12,13]. Similarly, other drugs used to treat BPH by reducing the smooth muscle tone include α-blockers (terazosin, doxazosin, tamsulosin, and alfuzosin) and PDE-5 inhibitors (PDE-5-Is) (Sildenafil, tadalafil, lodenafil, udenafil, and mirodenafil) [14,15]. The primary limitation of the use of these drugs is their side effects—for example, 5-α reductase inhibitors and α-blockers may cause ejaculatory dysfunction (ED), while PDE-5-Is can lead to anxiety and depression [16,17,18]. If medical management fails, there are various invasive surgical procedures, such as transurethral resection of the prostate and simple prostatectomy, that are considered gold-standard surgical interventions for treating BPH. These invasive surgical procedures are not without risks and have an association with perioperative complications such as bleeding and clot retention [19].

The paracrine hormone oxytocin (OT) is considered a potential novel target for the treatment of BPH. The role of OT is well-defined within the female reproductive tract, where it causes contractions of the uterus after binding to the oxytocin receptor (OXTR). OXTR then couples with G-protein coupled receptors, resulting in an increase in the intracellular calcium level. This increased intracellular calcium level is responsible for the initiation of myometrial contractions [20]. OT also causes contraction of myoepithelial cells, resulting in milk ejection from the myoepithelial alveoli through the milk ducts and towards the breast nipple [21,22,23,24]. In men, OT also contributes to the regulation of the male reproductive tract [25]. OT is locally produced in the prostate, testis, epididymis, pancreas, thymus, adipocytes, and kidney [26,27,28]. OT can further act on the prostate via the regulation of testosterone and DHT, resulting in an increase in contractility because of an increase in the number of epithelial and stromal cells within the prostate [26].

Therefore, the main objectives of the current study were (i) to evaluate the role of OT in prostate contractions and (ii) to assess the role of OT receptor antagonists (atosiban, cligosiban and ß-Mercapto-ß,ß-cyclopentamethylenepropionyl) on both spontaneous and OT-induced prostate contractions in the various *rat* age groups, i.e., a combination of two young (7–8 weeks and 10–12 weeks) and two older (16 weeks and 7–9 months) groups.

## 2. Materials and Methods

### 2.1. Animal Ethics

The prostate tissue from Sprague-Dawley (SD) male *rats* was scavenged at the animal housing located at Monash Institute of Pharmaceutical Sciences (MIPS), Parkville, and Monash Animal Research Platform (MARP), Clayton, Australia. The SD *rats* used in the organ bath study were a combination of young (7–9 weeks, 10–12 weeks) and older (7–9 months) age groups (*n* = 5, each group), while the immunohistochemistry study was performed on 7–9 weeks (young) and 16 weeks (older) age groups (*n* = 5, each group). *Rats* of young age were housed at Monash Institute of Pharmaceutical Sciences, Parkville, Australia, and those of older age at Monash Animal Research Platform Clayton, Australia, with a 12 h light/dark cycle and free access to food and water. All experiments were conducted according to guidelines and regulations required for animal care, and ethical approval was granted by the ethics committee under reference numbers MIPS-26791 and MARP-00000, respectively.

### 2.2. Tissue Collection

*Rats* were placed in a CO_2_ chamber and euthanised by CO_2_ inhalation. An incision in the abdomen was made along the midline to expose the whole prostate, which was then carefully dissected under binocular microscopy using fine surgical tools to isolate a ventral lobe from the multilayered lobe of the prostate. This ventral lobe was then divided into two equal halves and maintained in a minimal essential medium (size = 500 mL; *n* = 5; catalogue number = 51200038) (Thermo Fisher Scientific, Waltham, MA, USA).

### 2.3. Reagent Preparation

Powdered oxytocin (Sigma Aldrich, St. Louis, MO, USA), atosiban (Sigma Aldrich, St. Louis, MO, USA), and ß-Mercapto-ß,ß-cyclopentamethylenepropionyl (Sigma Aldrich, St. Louis, MO, USA) were dissolved in mili-Q water, while cligosiban (MedChem Express, St Lucia, QLD, Australia) was dissolved in DMSO (dimethyl sulfoxide) to form a stock solution of 10^−2^ M. This stock solution was then diluted further in mili-Q water to form a serial set of dilutions (10 pM, 100 pM, 1 nM, 10 nM, 100 nM, 1 µM). These serial dilutions were then incubated in an in vitro organ bath containing Krebs-Henseleit solution [Krebs-Henseleit solution, pH 7.4 (mM: NaCl 118.1, KCl 4.69, KH_2_PO_4_ 1.2, NaHCO_3_ 25.0, D (+) glucose 11.7, MgSO_4_·7H_2_O 1.1, CaCl_2_ 2.5)], to obtain the final concentration of a drug.

### 2.4. Organ Bath Studies

A typical organ bath was a double glass wall cylinder with a total volume of 10 mL, having the distal end tapered and clamped. The organ bath chamber was connected to the gas cylinder containing carbogen (95% O_2_ and 5% CO_2_). A fresh Krebs-Henseleit solution [(pH 7.4 mM: NaCl 118.1, KCl 4.69, KH_2_PO_4_ 1.2, NaHCO_3_ 25.0, D (+) glucose 11.7, MgSO_4_·7H_2_O 1.1, CaCl_2_ 2.5)] was placed in the chamber, bubbled with carbogen and heated to 37 °C throughout the experiment. Tissue strips were attached to the hook at one end and with a piece of string at the other end, which was then attached to the isometric force-displacement transducer that is present above the open end of the chamber. The transducer was connected to the PowerLab data acquisition system (AD Instruments, Bella Vista, NSW, Australia), which was connected to the computer system. The computer then recorded the tension created within the tissue using Chart Prov 5.5.6 (AD Instruments, Bella Vista, NSW, Australia). 

Organ bath studies were performed with an initial tension of 0.5–1 g applied to the prostate. The tissue was equilibrated for 60 min and observed for spontaneous contractions [29,30].

### 2.5. Spontaneous Contractions

After equilibration, tissue (prostate) was incubated with the concentrations of atosiban (AT) (1 µM), cligosiban (1 µM) and ß-Mercapto-ß,ß-cyclopentamethylene propionyl (ßMßßC) (1 µM). Tissue viability was tested using a high concentration of potassium chloride (20 mM), which was used before and after the experiment.

### 2.6. Statistical Analysis of Tension Recordings

The parameters of interest were the integral, which is the area under the curve (AUC), maximum value and frequency. These parameters were used previously to define spontaneous contractions [30,31,32]. Data was then extracted from the tension recordings using Chart Pro v 7.3.8, which was then exported to an Excel spreadsheet where they were averaged relative to the maximum percentage of potassium chloride (KCl; 20 mM). All the data were presented as mean ± SD. Statistical analysis was accomplished by student *t*-test, ordinary one-way ANOVA with the Tukey multiple comparisons test, with non-linear regression equation used within the prism for more accuracy [Graph Pad Prism for Windows version 9.0.1 (GraphPad Software, La Jolla, CA, USA)]. * *p* < 0.05 was considered significant.

### 2.7. Immunohistochemistry and Tissue Stains

Prostate tissues were first fixed in 10% NBF (neutral buffered formalin), followed by paraffin embedding performed at Monash Histology Platform. Paraffin-embedded tissue sections (4 µm thick) were then baked in an oven at 60 °C for 30 min, de-waxed with 100% xylene (3 × 2 min) and rehydrated with 100% ethanol (3 × 2 min). After rinsing in distilled water, the tissue sections were then washed with buffer (1× DAKO EnVision Flex Wash Buffer) (DAKO, Glostrup Kommune, Denmark, Cat # K8000) (2 × 5 min at room temperature). The next step was antigen retrieval, which was performed in DAKO PT Link containing low pH target retrieval solution (DAKO, Cat # S1699) at 98 °C for 30 min. The tissue sections were then washed with buffer (1× DAKO EnVision Flex Wash Buffer) (5 min at room temperature). Immunofluorescence labelling was performed on DAKO Autostainer Plus [Ft. Collins, CO, USA], which involves the following steps: (i) blocking for rat-on-mouse reactions with AffiniPure Fab Fragment *Donkey Anti-Mouse* IgG (H + L) (Jackson ImmunoResearch Labs Inc., West Grove, PA, USA, # 715-007-003) at a concentration of 200 µg/mL diluted in DAKO Protein Block Serum Free (DAKO, Cat # X0909) for 60 min at room temperature; (ii) buffer wash (1× DAKO EnVision Flex Wash Buffer) (2 × 5 min at room temperature); (iii) incubating the tissue sections with primary antibodies and isotype controls diluted in DAKO Antibody Diluent (DAKO, Cat # S0809) for 60 min at room temperature: Polyclonal *Rabbit*-anti-Oxytocin Receptor [1:50 dilution (20 µg/mL), Bioss, # B601086491], Monoclonal *Mouse-anti-Smooth Muscle Actin* [1:400 dilution (2.5 µg/mL), clone 1A4, DAKO # M0851], *Mouse* IgG isotype control: *Donkey Anti-Mouse* IgG (H + L) 1:400 dilution (20 µg/mL) (Jackson ImmunoResearch Labs Inc., Cat # 715-007-003), *Rabbit* IgG isotype control: *Rabbit* polyclonal Antibody IgG Isotype control 1:125 dilution (20 µg/mL) (CST # 3900S). Tissue sections were afterwards washed with buffer (1× DAKO EnVision Flex Wash Buffer) (3 × 10 min) and then incubated with the following secondary antibodies cocktail diluted in DAKO Antibody Diluent (DAKO, Cat # S0809) and incubated for 60 min at room temperature: (i) AffiniPure *Donkey anti-Rabbit* AF488 1:500 (1.5 µg/mL) (Jackson ImmunoResearch, # 711-545-152); (ii) AffiniPure *Donkey anti-Mouse* AF647 1:500 (1.5 µg/mL) (Jackson ImmunoResearch, # 705-605-151). Then wash again with buffer (1× DAKO EnVision Flex Wash Buffer) (3 × 10 min). Tissue sections were then counterstained with DAPI [1:10,000 dilution, Sigma Aldrich, Cat # D9542-10MG] for 15 min at room temperature, followed by a 5-min wash with distilled water. For the elimination of tissue autofluorescence, 0.3% Sudan Black B [Sigma, St. Louis, MO, USA, # 199664-25G] dissolved in 70% ethanol was applied for 1 min at room temperature, then washed for 5 min with distilled water. Lastly, coverslip the slides with Prolong Gold antifade reagent (Invitrogen, Waltham, MA, USA, Cat # P36934).

### 2.8. Analysis of IHC Experiments

All the prostate tissue sections (slides) were then scanned with VS120 Automated Slide Scanner at the Monash Histology Platform. For experimental slides, the exposure for each fluorophore was as follows: DAPI (4′,6-diamidino-2-phenylindole) (120 ms), AF 488 (1000 ms), and AF (647,590 ms). DAPI reveals nuclear staining, AF 488 for oxytocin receptors, and AF 647 for smooth muscle actin. All the images of the respective slides were captured with OlyVIA (version 2.9.1), analysed using a QuPath-0.3.2 (console) and the Fiji distribution of Image J [version 1.53t 24 August 2022 (upgrade)]. The average of the mean and standard error of the mean (mean ± SEM) were calculated and then transferred to an Excel spreadsheet. All the graphs were then plotted using Prism 9 [Graph Pad Prism version 9.0.1 for Windows (GraphPad Software, La Jolla, CA, USA)]. Statistical significance was performed using a two-tailed unpaired *t*-test after testing for a Gaussian distribution using the Shapiro-Wilk normality test. * *p* value < 0.05 denoted statistical significance.

## 3. Results

The mean ± SEM weights of whole prostates were 0.8 ± 0.012 g (7–9 weeks), 1.318 ± 0.104 (10–12 weeks), and 1.6 ± 0.08 g (7–9 months). The mean ± SEM weights of the whole rats were 320 ± 3.22 g (7–9 weeks), 401.8 ± 15.41 g (10–12 weeks), and 561.33 ± 11.74 g (7–9 months). 

### 3.1. Organ Bath Findings

#### 3.1.1. Atosiban Significantly Downregulated the Frequency of Spontaneous Contractions in the Prostate Tissue

**Figure 1:**
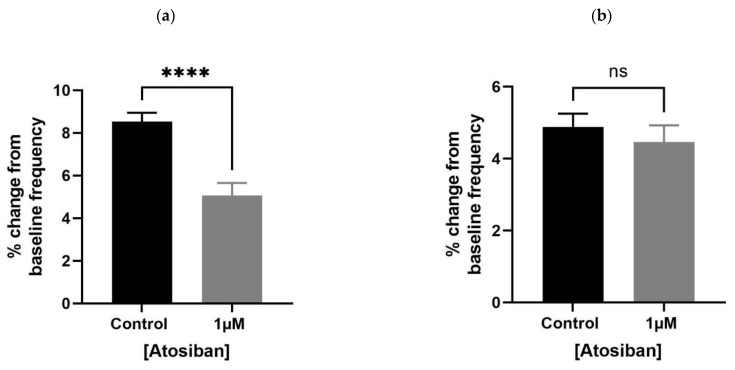
Effects of atosiban on the frequency of prostate movement. Application of atosiban (*n* = 5) (1 µM) significantly downregulated the % change in baseline frequency of spontaneous contractions in the prostate of young (**a**) but not older (**b**) *rats* (unpaired *t*-test, **** *p* < 0.0001) (**** denotes significant while ns shows non-significant effects).

**Figure 2:**
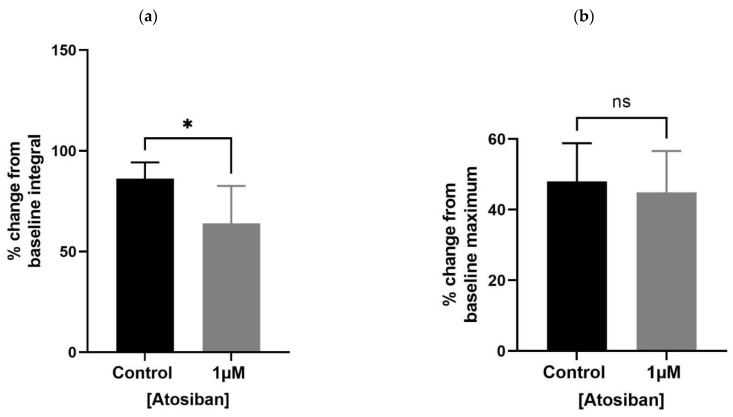

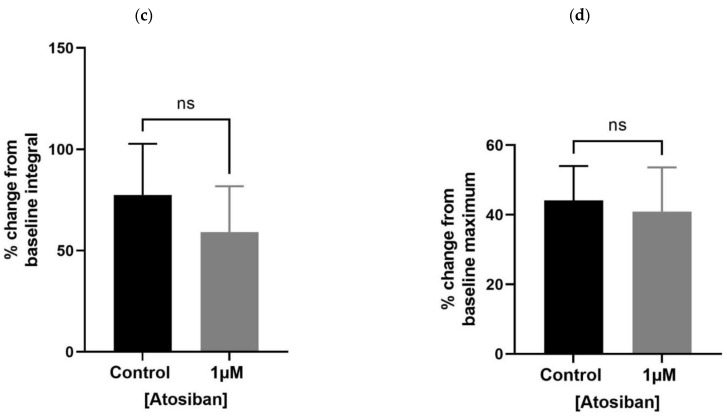
Effects of atosiban (1 µM) on prostate (spontaneous) contractions. Atosiban (1 µM) showed a significant effect on the baseline integral of spontaneous contractions in young (**a**) but not older (**c**) *rats*. However, some effect was observed on the baseline maximum parameters of spontaneous contractions in both young (**b**) and older (**d**) *rats*, calculated relative to the percentage of maximum concentration of potassium chloride (KCl; 20 mM) (unpaired *t*-test, *n* = 5, *p* ≥ 0.05) (* denotes significant and ns shows non-significant effects).

Atosiban (1 µM) significantly reduced the frequency of spontaneous contractions in the prostate of young (10–12 weeks) (Figure 1a) but not the older (7–9 months) (Figure 1b) *rats*. Also, atosiban showed a significant effect on the baseline integral of prostate contractions of young (Figure 2a) but not older (Figure 2c) *rats*. However, it revealed some effect (non-significant) on the baseline maximum parameter of prostate movements of both young (Figure 2b) and older (Figure 2d) *rats*, measured as a percentage relative to the percentage of the maximum concentration of potassium chloride (KCl; 20 mM).

#### 3.1.2. Cligosiban Achieved Significant Inhibition of Spontaneous Activity in Older *Rats*

**Figure 3:**
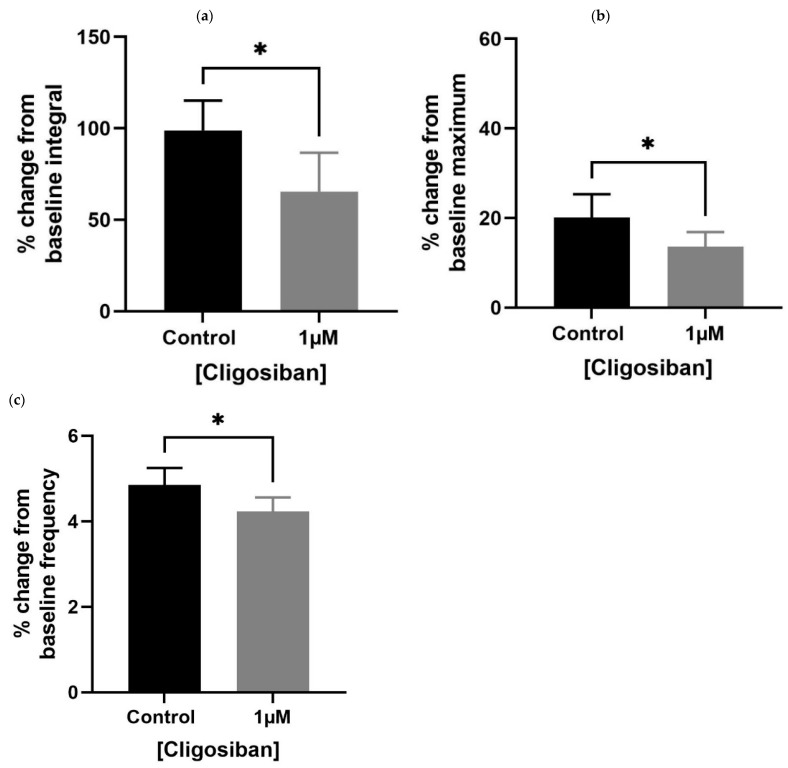
Administration of exogenous cligosiban (1 µM) significantly attenuated the % change in baseline integral (**a**), maximum value (**b**), and frequency (**c**) of spontaneous contractile activity within the prostate of older (7–9 months) *rats*, averaged relative to the percentage of maximum concentration of potassium chloride (KCl; 20 mM) (*n* = 5, unpaired *t*-test, * *p* < 0.05) (* denotes significant effect).

Older (7–9 months) *rat* prostates showed a significant effect in decreasing spontaneous contractions in response to cligosiban (1 µM). A decrease in all the parameters, i.e., baseline integral (Figure 3a), maximum value (Figure 3b) and frequency (Figure 3c), was observed, demonstrating the antagonistic effects of cligosiban on the prostate movements that are induced by the endogenous release of oxytocin (OT).

#### 3.1.3. β-Mercapto-β,β-cyclopentamethylenepropionyl1, O-Me-Tyr2, Orn8]-Oxytocin (ßMßßC) Significantly Reduced the Spontaneous Contractions in Older *Rats*

**Figure 4:**
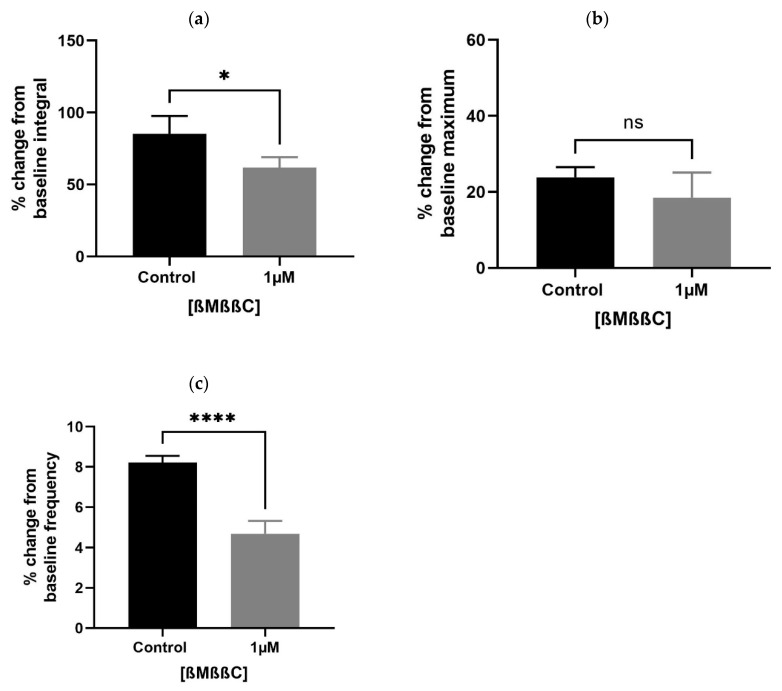
Effects of ßMßßC (1 µM) on smooth muscle (prostate) contractions. ßMßßC showed a significant effect in decreasing the % change in baseline integral (**a**) and frequency (**c**) of spontaneous contractile activity generated within the prostate of older (7–9 months) *rats*. However, some change (non-significant) was detected in the baseline maximum value (**b**) of spontaneous contractions (*n* = 5, unpaired *t*-test, * *p* < 0.05 and **** *p* < 0.0001) (* and **** denote significant while ns shows non-significant effects).

A lower concentration of ßMßßC (1 µM) significantly downregulated the baseline integral (Figure 4a) and frequency (Figure 4c), with some change (non-significant) in the maximum value (Figure 4b) of spontaneous contractions observed within the prostate of older (7–9 months) *rats*. These values were presented as a percentage that is calculated relative to the percentage of the maximum concentration of potassium chloride (KCl; 20 mM).

### 3.2. Immunohistochemistry (IHC) Findings

#### Oxytocin Receptor (OXTR) Is Widely Expressed within the Epithelial Compartment of Rat Prostate

**Figure 5:**
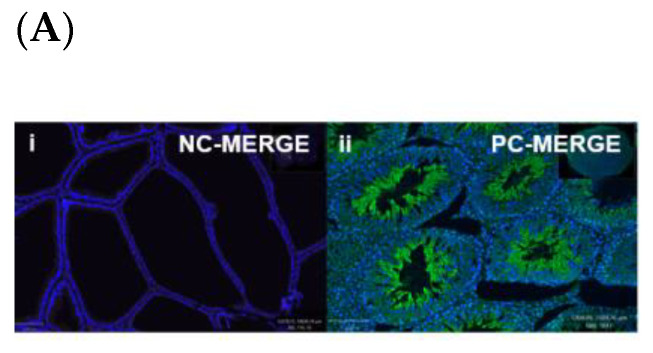

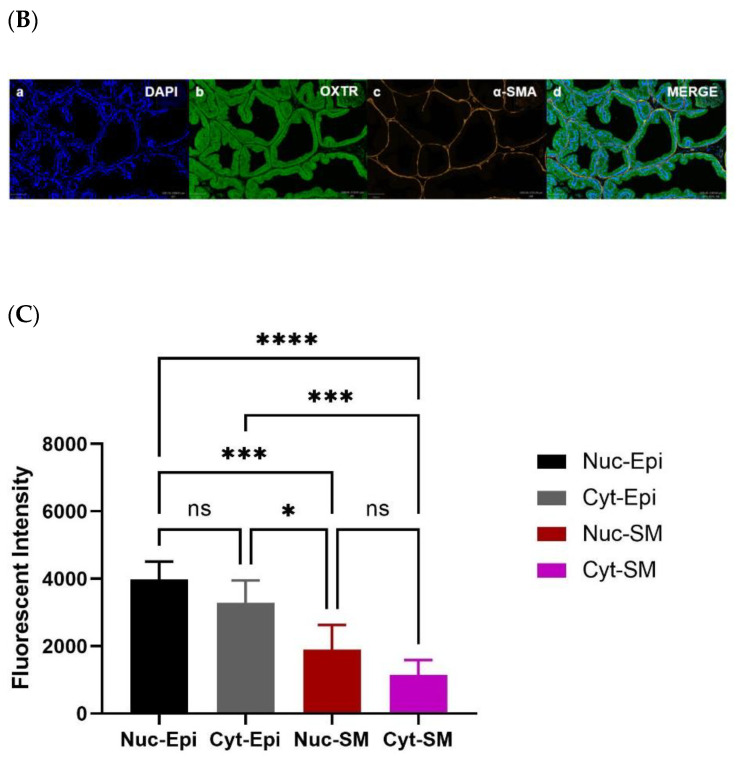
Immunofluorescence of a young *rat* prostate. Representative of double immunofluorescence (IF) staining for α-SMA and OXTR of negative control of a *rat* prostate (**Ai**), positive control (rat testis) (**Aii**). IF images (**Ba**) DAPI reveal nuclear staining, (**Bb**) highlight OXTR staining, (**Bc**) show actin staining and (**Bd**) merge image displays colocalisation of epithelial cells with OXTR within the prostate (scale bar = 100 µm). Graph (**C**) demonstrates differences in the intensity of OXTR staining between the nuclear and cytoplasmic regions of epithelial and smooth muscle cells (One-way ANOVA with Tukey’s multiple comparisons test, **** *p* < 0.0001) [NC-MERGE = Negative Control; PC-TESTIS = Positive Control Testis; DAPI = 4′,6-diamidino-2-phenylindole; OXTR = Oxytocin Receptor; α-SMA = Alpha Smooth Muscle Actin; MERGE = Merged Image; Nuc-Epi = Nucleus Epithelium; Nuc-SM = Nucleus Smooth Muscle; Cyt-Epi = Cytoplasm Epithelium; Cyt-SM = Cytoplasm Smooth Muscle] (*, *** and **** denotes significant while ns shows non-significant effects).

**Figure 6:**
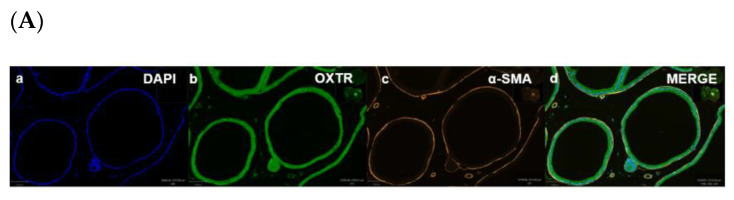

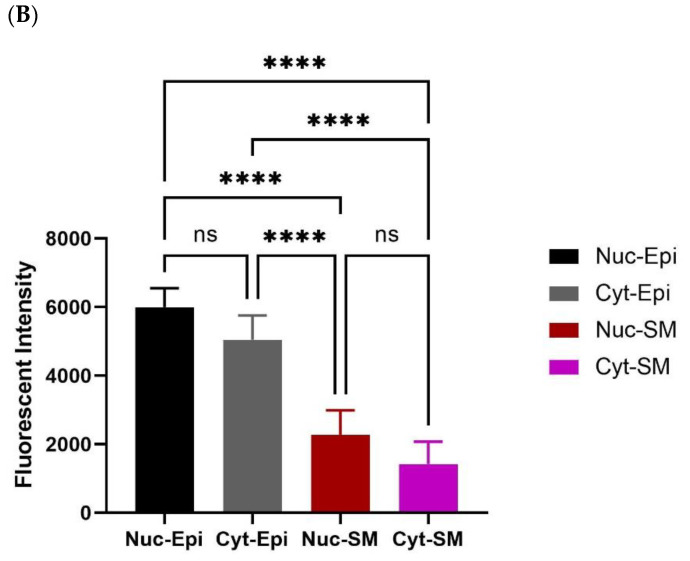
Measurement of oxytocin receptor (OXTR) within old *rat* prostate. Immunofluorescence staining (**Aa**) DAPI for nuclear staining, (**Ab**) OXTR staining, (**Ac**) actin staining, and (**Ad**) merge image reveals colocalisation of OXTR with epithelial cells (scale bar = 100 µm). Graph (**B**) compares nuclear and cytoplasmic staining of OXTR of epithelium and smooth muscle cells (One-way ANOVA with Tukey’s multiple comparisons test, **** *p* < 0.0001). [DAPI = 4′,6-diamidino-2-phenylindole; OXTR = Oxytocin Receptor; α-SMA = Alpha Smooth Muscle Actin; MERGE = Merged Image; Nuc-Epi = Nucleus Epithelium; Nuc-SM = Nucleus Smooth Muscle; Cyt-Epi = Cytoplasm Epithelium; Cyt-SM = Cytoplasm Smooth Muscle] **** denotes significant while ns shows non-significant effects).

**Figure 7:**
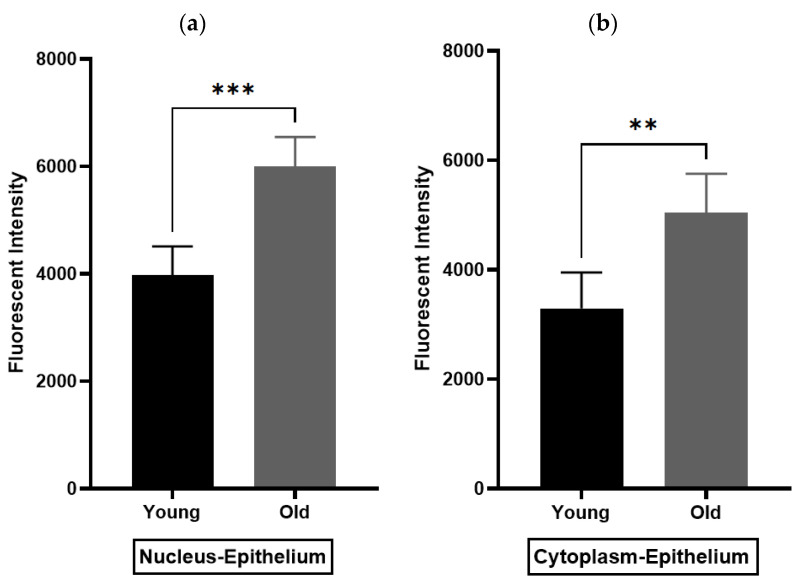

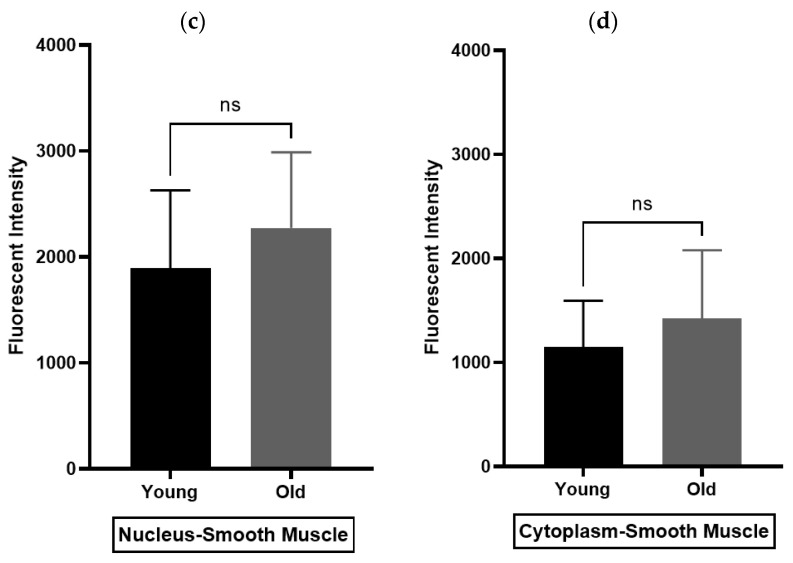
Immunohistochemistry analysis of rat prostate. (**a**,**b**) compare the density of nuclear and cytoplasmic staining of oxytocin receptor (OXTR) within the epithelial compartment, while (**c**,**d**) the smooth muscle compartment of young and older *rat* prostate (unpaired *t*-test) [Nuc-Epi = Nucleus Epithelium; Cyt-Epi = Cytoplasm Epithelium; Nuc-SM = Nucleus Smooth Muscle; and Cyt-SM = Cytoplasm Smooth Muscle] (** and *** denotes significant while ns shows non-significant effects).

Immunohistochemistry (IHC) was performed to demonstrate the existence and distribution of oxytocin receptor (OXTR). OXTR expression was detected in both the epithelium and stroma (smooth muscle cells) of both age groups (young = Figure 5A,B) (old = Figure 6A). The graphs indicate that the intensity of OXTR staining was increased within the epithelial compartment of both young (7–9 weeks) (Figure 5C) and older (16 weeks) (Figure 6B) *rats*. However, a weak OXTR staining was observed in the smooth muscle cells. Also, the intensity of both nuclear and cytoplasmic OXTR staining increased with age within both the epithelial [Figure 7a,b] and smooth muscle [Figure 7c,d] regions, with a larger intensity observed in the epithelium of older *rats* (Figure 7a,b). Overall, the prostate epithelium of both age group *rats* showed consistent OXTR staining, indicating its potential indirect effect on the underlying smooth muscles via the paracrine mediator-like oxytocin.

## 4. Discussion

The present study explored the role of oxytocin (OT) in prostate function and is the first research that compares the receptor-mediated effects of atosiban, cligosiban and ß-Mercapto-ß,ß-cyclo pentamethylene propionyl (oxytocin receptor antagonists) on spontaneous as well as OT-induced contractions within the prostate from different age group *rats*. 

During this research, a combination of two young (7–9 and 10–12-weeks) and two older (14–16 weeks and 7–9 months) age group *rats* were used due to the following reasons: (i) As benign prostatic hyperplasia (BPH) is a disease of an ageing population, it is worth investigating the OT effects in young as well as older *rats* as old rats may show OT-related changes [31]; (ii) There are age-related changes already noticed in the smooth muscle contractility within the prostate of *guinea pigs*; (iii) It was also observed that OT has a similar effect in both *rats* and *humans* in increasing prostatic contractions [33].

Oxytocin (OT) is a nonapeptide found in most of the vertebrates. It has a very similar structure to the arginine vasopressin (AVP) family, differing only in the third and eighth position of amino acids [34,35]. Thus, due to the similarity in its structure, its receptors (OXTR and AVPR) are similar as well [36]. Atosiban is a competitive arginine vasopressin/oxytocin receptor (AVP/OXTR) antagonist that is most commonly used in Europe for the treatment of preterm labour, with minor side effects reported such as nausea, hyperglycaemia, headache, dizziness, and palpitations [37,38]. Its mechanism of action is via the dose-dependent inhibition of OT-mediated increase in intracellular calcium level, resulting in the closing of voltage-gated calcium channels to prevent the entry of calcium. On the other hand, cligosiban and ßMßßC have not been used clinically. Recent studies mentioned that cligosiban, a highly selective non-peptide OT receptor antagonist, inhibited apomorphine-induced ejaculation in *rats*. During this study, the central nervous system (CNS) penetration of cligosiban was assessed via measurement of cerebrospinal fluid (CSF) and plasma drug concentrations following intravenous (I/V) infusion in two different types of *rat* models, i.e., the electromyography model of ejaculatory physiology and the OT-mediated CNS neuronal firing model. In the electromyography model, an I/V bolus of cligosiban (0.9 mg/kg) resulted in a decrease in bulbospongiosus burst pattern and contraction amplitude correlated with ejaculation. However, the same I/V bolus of cligosiban (0.9 mg/kg) reduced the OT-mediated response in the nucleus of tractus solitarius in the CNS neuronal firing model [39]. Contrary to this study, a recent clinical trial showed that cligosiban failed to prove its efficacy in the treatment of premature ejaculation [40]. On the other hand, ßMßßC, an oxytocin receptor antagonist, was recently used to block OT receptors, resulting in a decrease in intravesical pressure (IVP) within female *rats’* bladders, suggesting that it may be used to treat bladder dysfunction, which is highly prevalent in women [41].

The present organ bath study used the same concentrations (1 µM) for all the antagonists because of their similar potency to OXTR. This study showed that the peptide atosiban (AT) significantly attenuated the frequency and integral (AUC) parameters of spontaneous contractions within the prostate of young *rats*, with cligosiban and ßMßßC in the older *rats*, indicating age-related differences in the drug’s efficacy and tissue physiology. Overall, the effects of these antagonists on prostate contractility were small (% change < 10% in all parameters). However, these findings support our hypothesis that endogenous oxytocin (OT) may contribute to increasing prostatic movements. Based on these findings, it might be valuable to consider AT as a new treatment option for BPH in young *rats*, while CLIGO and ßMßßC, though still under trial, could be considered promising candidates in the older age population.

Additionally, our in vitro organ bath studies showed that aged *rats* exhibited a trend in the increase of the integral parameter as OT concentration was increased, with no change in the parameters such as maximum and frequency observed. These findings were similar to those reported by Bodanszky et al. (1992), who found an increasing effect of OT on spontaneous contractions within the *rat*, *guinea pig*, *dog* and *human* prostate [34]. Likewise, an invitro organ bath study performed by Lee et al. (2021) observed that atosiban at a concentration of 300 nM significantly attenuated the frequency (34.9  ±  7.8%), basal tension (9.52  ±  1.42%), and amplitude (34.7  ±  6.9%) of spontaneous contractions within the *human* prostate (*n* = 15; age range = 53–75 years) [33]. Furthermore, these spontaneous events were visualised within the *guinea pig* prostate via standard intracellular microelectrode recording techniques. Under an electron microscope, a subpopulation of cells called c-Kit positive cells was detected within the interstitial space between the glandular layer and the stroma, having a similar role to that of interstitial cells in the gut in which they act as a pacemaker resulting in slow-wave activity. This slow-wave activity is myogenic in origin, and its frequency is increased by excitatory agents such as phenylephrine, high potassium and histamine. It is believed that this slow-wave activity is of similar frequency and half-duration amplitude as that of spontaneous contractions [31]. Contrary to this, young *rats* in the present study showed no increase or decrease in integral, maximum, and frequency parameters as the OT concentration was increased. These results were similar to the in vitro organ bath study conducted by Gupta et al. (2008), who measured the OT-induced isometric tension within the prostate of *rats* and *rabbits* utilising standard tissue bath technique and noticed no such OT effect [42]. 

The oxytocin receptor (OXTR) belongs to a member of the rhodopsin-type (class 1) family of GPCRs that mainly respond to a neurohypophyseal hormone called oxytocin (OT) [36,43]. These GPCRs are seven transmembrane proteins that are located within the cell membrane and nucleus of a cell [44,45,46,47]. A study conducted by Herbert et al. (2007) reported that these OXTRs are located within and outside the caveolae, which are invaginations of the plasma membrane [48]. Some studies also noticed that OXTR is present in the nucleus of human osteosarcoma and breast cancer cell lines [49]. However, the functional significance of OXTR location within the nucleus is not clear. However, it is believed that this nuclear localisation is *Arrb-* and *Tnpo1-dependent* [50]. There are also other nuclear-localised receptors like the estrogen receptor, progesterone receptor, thyroid receptor, chemokine receptor 2 (CCR2), and cysteine (C)-X-C receptor 4 (CXCR4). Hormones such as estrogen, progesterone and thyroid cross the cell membrane and interact with the receptors present in the nucleus. This then results in the activation of nuclear receptors that mainly regulate gene transcription, thus helping in controlling a variety of biological processes, including cell development, metabolism, proliferation and reproduction [51].

There is only one type of OXTR identified within the prostate, and it is a G-protein coupled receptor type [52,53], and its existence varies from species to species [53]. For example, they are expressed in both epithelial and smooth muscle cells of the human prostate [54], epithelial cells lining the prostatic acini of *dogs*, *possums* and *rats* [26,53], and basal layers of the secretory epithelium in the prostate of *marmoset monkeys* [55]. Also, immunoreactivity for OXTR was observed in the epithelial compartment in the hyperplastic prostate [56], with the stromal part in the normal *human* prostate [57]. This existence and distribution of OXTR were recently studied by Lee et al. (2021), who conducted an immunohistochemistry (IHC) study on the *human* prostate utilising a cohort of older patients (>53 years old). During this study, it was found that OXTR was expressed in both epithelial and stromal cells. To find out if OXTR was colocalised with smooth muscle cells, double immunofluorescence staining was performed for OXTR and α-Smooth Muscle Actin (α-SMA). It was reported that OXTR was colocalised with smooth muscle cells within the human prostate, indicating OT is a modulator of prostate contractility. Thus, this study provides a rationale for further examining the use of OXTR antagonists for the medical treatment of BPH [54]. 

The current study is the first that quantifies the specificity and intensity of the oxytocin receptor (OXTR) within the epithelial and stromal regions of the *rat* prostate. Our immunofluorescence staining revealed that these OXTRs are mostly expressed within the epithelial compartment of both young and older *rats* and are mainly located within the nucleus of a cell. These findings support our organ bath findings and suggest that the prostate epithelium may have an indirect influence on underlying contractile (smooth muscle) cells via the paracrine hormone oxytocin. This then results in an increase in smooth muscle contractions within the prostate. These findings were similar to those reported by Li et al. (2018), who examined the effects of hyperplastic prostate on OXTR expression within the testosterone estradiol-induced *rat* model (*n* = 15; 12-week-old *Wistar rats*) and human hyperplastic prostatic specimens (*n* = 9; mean age, 67.7 ± 2.1 years) undergoing cystoprostatectomy for infiltrating bladder cancer without prostate infiltration. During this study, they utilised the immunohistochemistry technique (IHC) and observed that OXTR is present in the epithelium in *rats* and the stroma in the *human* prostate, indicating BPH is an epithelial disease in rats with stromal disease in *humans* [53]. Contrary to this, an IHC study conducted by Einspanier and Ivell (1997) observed weak staining of OXTR in the basal layers of the secretory epithelium of the prostate of *marmoset monkeys* (*n* = 6; >18 months old) [55]. Furthermore, Stadler et al. (2021) conducted a chromogenic immunohistochemistry study on tissues collected from *men* undergoing transurethral resection of the prostate (TURP) for benign prostatic hyperplasia (BPH) (*n* = 6) and prostatectomy for prostate cancer (*n* = 6). She noticed colocalisation of OXTR with the smooth muscle cells and weak staining of OXTR in the epithelium of the *human* prostate [28]. Additionally, an immunocytochemistry study led by Herbert et al. (2007) noticed the age-related differences in OXTR staining within the human prostate (*n* = 4), i.e., weak staining in 9- and 18-year-old young *men* and strong staining in 28- and 33-year-old *men* and BPH patients (*n* = 10; age range = 54–75 years) [48]. Thus, there are differences noted between the present study and those reported in the literature. However, all these studies used the same immunohistochemistry (IHC) technique. But, these differences observed in the intensity of OXTR staining could be due to species variability as well as age differences in the current study. We used the prostate from different age groups of SD *rats* (7–9 week young and 16-week old) *rats*, while Einspanier and Ivell (1997) used *marmoset monkeys* (>18 months old) [55], Stadler et al. (2021) utilised BPH and prostate cancer *human* specimens [28], Herbert et al. (2007) conducted this study on young healthy (9- and 18-year-old; and 28- and 33-year-old *men)* and BPH prostate tissue (age range = 54–75 years) [48], and Lee et al. (2021) collected *human* prostate tissue from a cohort of older patients (>53 years old) [54]. Therefore, based on the current findings and the findings highlighted in the literature, it is important to further investigate the microenvironment within the prostate to better understand the pathophysiology of LUTS/BPH. 

There are also other factors, such as hormones (testosterone), that may interfere with oxytocin signalling. For example, a couple of studies [Lee et al., 2021; Ellem and Risbridger (2009)] reported that such changes in the intensity of OXTR staining could be due to an age-mediated decrease in the testosterone level while the oestrogen level remains stable [54,58]. Therefore, a future direction could be to compare the intensity of OXTR staining between men treated with 5-α-reductase inhibitors and those not (control), as this treatment may cause hormone (testosterone) imbalance that may interfere with oxytocin signalling [54].

A limitation of our study is that we did not further investigate the effects of cligosiban and beta-Mercapto-beta, beta-cyclo pentamethylene propionyl (ßMßßC), the oxytocin receptor antagonists on spontaneous contractions within the prostate of young *rats*. A future direction should be to evaluate the effects of oxytocin receptor antagonists on the prostate movements that occurred due to the endogenous release of oxytocin. A second limitation associated with this study is to determine the expression of the oxytocin receptor gene within the prostate utilising real-time PCR (RT-PCR).

## 5. Conclusions

In conclusion, BPH is known to be a highly prevalent disease of ageing men, with limited drug therapies available. Our in vitro organ bath study highlighted the importance of atosiban, an oxytocin receptor antagonist, in attenuating the frequency and integral parameters of spontaneous contractions in young *rats* and cligosiban and ßMßßC in older *rats*. Additionally, these organ bath findings were supported by an immunohistochemistry (IHC) study that demonstrated consistent staining of OXTR within the epithelial compartment of the prostate of both young and older *rats*. Thus, the magnitude of the effects showed by OT proposes that OXTR overexpression might be involved in the pathophysiology of BPH. So, targeting OXTR utilising oxytocin receptor antagonists can be clinically effective in the treatment of lower urinary tract symptoms associated with benign prostatic hyperplasia (LUTS/BPH).

## Data Availability

Data is unavailable due to privacy or ethical restrictions.
